# The Impact of Age on Mobile Technology Use During the COVID-19 Pandemic

**DOI:** 10.1093/geroni/igab046.3645

**Published:** 2021-12-17

**Authors:** Yi Lin, Graham Rowles

**Affiliations:** University of Kentucky, Lexington, Kentucky, United States

## Abstract

The COVID-19 pandemic led to quarantines and mandatory spatial distancing; people of all ages were encouraged to use technologies instead of actual human contact as part of COVID-19 prevention. The promotion of mobile applications (apps) during the pandemic influenced mobile technology use behavior. This study explored age differences in mobile technology use during the COVID-19 pandemic. A pilot-tested survey was distributed using online survey software. Persons surveyed were 35 years of age or older, currently living in the United States of America with experience using mobile technology. Survey questions pertained to mobile technology use frequency and factors influencing the decision to use mobile technology. The nationwide response included 1212 individuals. The average age of participants is 56.12±12.26 years old (female: male = 1.24:1). Responses were categorized participants into three age groups, 35 to 49, 50 to 64, and 65 or older. Daily mobile technology use frequency increased significantly (p<0.01) for all groups during the COVID-19 pandemic, with participants 35 to 49 having a significantly higher (p<0.01) use frequency than other groups. Regarding factors influencing the decision to use mobile technology, 64.1% of respondents aged 50 to 64 identified the necessity of using this technology during the pandemic as a significant factor, and 64.0% of participants in age 65 or older reported that the availability of functions on mobile devices is critical. Overall, the COVID-19 pandemic led to a significant increase in mobile technology use with people in different age groups differentially valuing the factors that affected their user behavior.

